# One-Pot Hydrothermal Synthesis of mSiO_2_-N-CDs with High Solid-State Photoluminescence as a Fluorescent Probe for Detecting Dopamine

**DOI:** 10.3390/nano13232989

**Published:** 2023-11-21

**Authors:** Xiaogao Guan, Xianzhu Xu, Yanli Wu, Ruchun Yang, Xi Chen, Fangfang Kong, Qiang Xiao

**Affiliations:** 1 Jiangxi Key Laboratory of Organic Chemistry, Jiangxi Science and Technology Normal University, Nanchang 330013, China; 18270143800@139.com (X.G.); ouyangruchun@163.com (R.Y.); chenxi2016@email.ncu.edu.cn (X.C.); xiaoqiang@tsinghua.org.cn (Q.X.); 2 College of Life Science, Jiangxi Normal University, Nanchang 330031, China; 9901189@163.com

**Keywords:** fluorescent probe, detection of dopamine, carbon dots, mesoporous silica, solid-state photoluminescence

## Abstract

An effective fluorescent probe (mSiO_2_-N-CDs) was prepared by embedding N-CDs into mesoporous silica via a simple one-pot hydrothermal reaction and applied to the detection of dopamine (DA). Mesoporous silica not only provided a skeleton to prevent the aggregation of N-CDs but also a medium for the centrifugal collection of N-CDs, avoiding the need for dialysis and freeze-drying. The formation process, phase composition, morphology, and luminescence properties of the composite were studied in detail. The synthesized mSiO_2_-N-CDs possessed spherical morphology, a smooth surface, and a diameter of approximately 150 nm. The fluorescence results indicated that mSiO_2_-N-CDs emitted intense blue color fluorescence at 465 nm under the optimal excitation of 370 nm. Because the mesoporous silica effectively inhibited the self-quenching caused by the aggregation of N-CDs, the quantum yield of solid mSiO_2_-N-CDs powder reached 32.5%. Furthermore, the emission intensity of the solid mSiO_2_-N-CDs remained constant for 28 days. The good sensitivity and selectivity of mSiO_2_-N-CDs for DA enabled the establishment of a rapid, simple, and sensitive DA detection method. The linear range was 0–50 µM and the limit of detection was calculated to be 107 nM. This method was used for the determination of DA in urine, with recovery rates ranging between 98% and 100.8%. In addition, the sensing mechanism was characterized by fluorescence lifetime decay and UV–VIS spectral analysis.

## 1. Introduction

As a key neurotransmitter in the human hypothalamus and pituitary gland, dopamine (DA) plays a vital role in regulating the nervous system, cardiovascular system, kidney, hormone secretion system, etc. DA imbalances can lead to psychiatric disorders (such as Parkinson’s disease, epilepsy, depression, anxiety disorders, [1,2,3,4] etc.). Therefore, it is very important to detect DA and its precursors (such as tyrosine and phenylalanine) accurately and sensitively for the diagnosis of nervous system diseases.

Technologies for the rapid and accurate detection of DA and its precursors are becoming increasingly important in the medical field. To date, various analytical methods for detecting DA and its precursors—such as high-performance liquid chromatography (HPLC) analysis [5,6], electrochemical sensors [7,8,9,10,11,12,13,14,15,16] and fluorescence sensors [17]—have been developed. Although HPLC is accurate, efficient, and sensitive, the need for expensive instruments and specialized operators has limited its application in the field of rapid DA detection. In recent years, electrochemical technologies have gained widespread interest because of their good sensitivity and low cost. However, the selectivity of electrochemical sensors is easily interfered with by other biological molecules, which limits their application in DA detection. For example, DA, ascorbic acid, and uric acid usually coexist in body fluids and the oxidation peak potential of the three substances is similar, making it difficult to use electrochemical methods to determine three substances [18,19]. Due to the significant advantages of fluorescence analysis, such as high sensitivity, strong selectivity, and ease of use, studies in recent years of the detection of DA using fluorescence methods have received increasing attention and many kinds of fluorescent sensors have been applied to the determination of DA [20,21,22,23,24,25,26,27]. For example, Zhou et al. [21] synthesized gold–silver nanoclusters for the detection of DA. Xu et al. [22] constructed a sensing platform for the detection of DA based on thiol-modified monolayer MoS_2_ nanosheets. Ling et al. [24] proposed a fluorescent probe based on water-soluble Tb^3+^-doped NaGdF_4_ nanomaterials for the dual-signal detection of DA.

As a new class of fluorescent nanomaterial, carbon dots (CDs) have gained great interest because of their good optical properties, excellent biocompatibility, low toxicity, easy surface modifiability, and excellent water solubility. Because DA can significantly quench the fluorescence of CDs, some studies have used them to perform qualitative and quantitative analysis and detection of DA [27,28]. Recently, there has been increasing research attention on fluorescent DA-sensing platforms based on CDs [27,28,29]. For example, Wang et al. [27] constructed a DA sensor using CDs doped with sulfur and nitrogen (S-N-CDs). Zhuo et al. [28] fabricated Fe-doped CDs for dopamine-sensing applications.

Despite these efforts, however, several key challenges for CD-based fluorescence sensors remain. At present, CD preparation processes are still relatively complex, requiring tedious operations such as dialysis and freeze-drying [30]; thus, large-scale synthesis of CDs is challenging [31]. Additionally, agglomeration of CDs in concentrated solutions or pure CD solids often leads to a decrease in luminescence, which is defined as aggregation-caused quenching (ACQ) and is another major factor limiting their application [32,33]. Some studies have shown that dispersing CDs in porous matrices (such as zeolite [34], carbonaceous porous materials [35,36], porous metal compounds [37,38], metal–organic frameworks [39,40], and mesoporous silica [41,42]) is an effective way to overcome these shortcomings. Among them, mesoporous silica has a porous structure and its pore size range can be controlled (2 to 50 nm), making it compatible with various sizes of CDs. In addition, it possesses good biocompatibility, high chemical stability, and easy surface functionalization. In recent years, research into the preparation and application of mesoporous silica CDs composite materials has aroused widespread interest among researchers [43,44,45,46]. For example, Liu et al. [43] established a fluorescence detection platform for Cr(VI) detection in water by anchoring mesoporous silica nanospheres with B-doped CDs. Chang et al. [44] obtained a fluorescent composite material (N-CD-SBA-15) with good solid-state fluorescence and excellent stability through simple ultrasonic treatment of a mixture of N-CDs and SBA-15. Wu et al. [46] developed a fluorescent sensor based on mesoporous silica-coated N-CDs for sensitive detection of picric acid. However, to our knowledge, there are currently no reports of embedding CDs into mesoporous silica to construct a fluorescent probe for the detection of DA.

This work aimed to construct a fluorescent probe taking mesoporous silica (mSiO_2_) as the skeleton and N-CDs as the fluorescent component and to explore its application in the detection of DA. Citric acid (CA) and urea (UR) were used as carbon and nitrogen sources, respectively. N-CDs were generated in situ in the pore channels of mesoporous silica by a one-pot hydrothermal process. The preparation process was facile and low-cost, suitable for large-scale synthesis. Based on the prepared mSiO_2_-N-CDs with stable and high solid-state photoluminescence and being highly sensitive to DA, a fast, simple and sensitive DA detection method was established (with detection limit of 107 nM, linear range of 0–50 µM).

## 2. Materials and Methods

### 2.1. Chemicals and Instruments

The chemical reagents and apparatus are described in detail in the Supplementary Materials.

### 2.2. Preparation of Mesoporous Silica Spheres (mSiO_2_)

The preparation of silica was slightly modified according to previous reports [43,47]. Triethanolamine (30 mg) was mixed with water (15 mL) under stirring at 80 °C. Subsequently, cetyltrimethylammonium bromide (CTAB, 190 mg) and sodium salicylate (NaSal, 84 mg) were added to the solution. After stirring for 1 h, tetraethyl orthosilicate (TEOS, 2 mL) was injected drop by drop into the above solution. After continuous stirring for 2 h, the reaction solution was separated by centrifugation for 20 min (10,000 rpm) to yield a white precipitate. Eventually, the resulting precipitate was ultrasonically dispersed in an HCl/CH_3_OH solution (1:10 *v*/*v*, 100 mL), and the organic template was removed by continuous reflux at 80 °C for 6 h. The template removal process was repeated once. The mixture was centrifuged (10,000 rpm) for 20 min and the precipitate was cleansed repeatedly with deionized water and ethanol, then dried at 60 °C in an oven. The obtained mSiO_2_ was characterized before further use.

### 2.3. Preparation of mSiO_2_-N-CDs

Citric acid (CA, 2.1 g) and urea (UR, 1.8 g) were added to deionized water (50 mL) and the mixture was stirred until a transparent solution formed. Then, the obtained mSiO_2_ (358 mg) was ultrasonically dispersed in this solution and the mixture was transferred to a hydrothermal reactor and kept at 160 °C in an oven for 12 h. After cooling, the mixture was centrifuged (10,000 rpm) for 20 min and the precipitate was cleansed repeatedly with deionized water and ethanol. It was then placed in a vacuum drying chamber for 24 h at 40 °C to generate mSiO_2_-N-CDs. The obtained product was stored at 4 °C for further applications.

### 2.4. Preparation of N-CDs

N-CDs were prepared by the one-pot hydrothermal method: CA (2.1 g) and UR (1.8 g) were added to deionized water (50 mL), then transferred into a 100 mL autoclave and maintained at 160 °C for 12 h. After cooling down, the mixture was separated by centrifugation (10,000 rpm, 10 min). The supernatant was collected and purified through a dialysis process (10,000 MWCO, 48 h), then further freeze dried to obtain solid N-CDs.

### 2.5. DA Detection Method

The detection of DA was analyzed in phosphate buffer (PBS, pH = 7.4). The emission spectra of the mixture containing mSiO_2_-N-CDs (0.25 mg/mL) and various concentrations of DA (0–50 µM) were tested under the optimal excitation of 370 nm. All samples were tested in triplicate.

To investigate the selectivity of mSiO_2_-N-CDs for DA detection, fluorescence intensity tests were conducted with the addition of interfering ions such as Ca^2+^, Zn^2+^, Na^+^, K^+^, Mg^2+^, and interfering biomolecules such as CA, UA, cysteine, glucose, galactose, fructose, epinephrine, and norepinephrine. The above interfering agents and DA in the same concentration (50 µM) were added to the mSiO_2_-N-CDs solution separately, and their emission intensities were compared with that of the solution without interfering substances. All experiments were performed in triplicate.

### 2.6. Real Sample Detection

In order to verify the feasibility of this method, the urine of healthy volunteers was used as the analysis sample and standard addition experiments were conducted. The urine was centrifuged at 10,000 rpm for 10 min to remove the sediments, and the supernatant was diluted 10-fold with phosphate buffer solution (PBS, pH = 7.4). The urine samples were spiked with DA (0.5, 1, 5, 40 µM), and DA concentration was determined by the proposed method. All experiments were conducted in triplicate.

## 3. Results and Discussions

### 3.1. Formation Process, Morphology and Composition of mSiO_2_-N-CDs

**Scheme 1:**
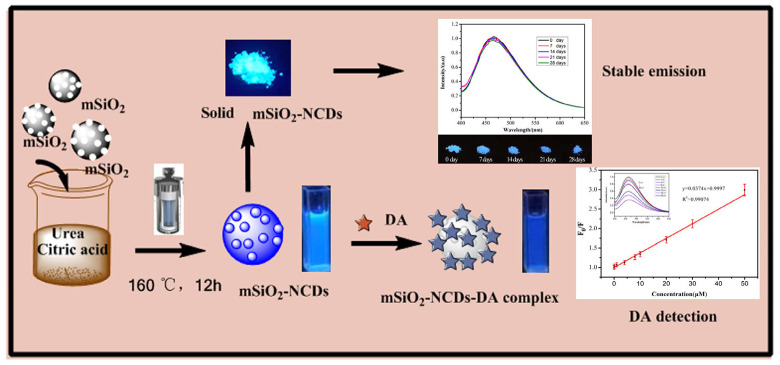
Synthesis diagram of mSiO_2_-N-CDs and their application in DA detection.

A novel fluorescent probe for DA detection (mSiO_2_-N-CDs) was designed and synthesized using mesoporous SiO_2_ as the framework and N-CDs as the fluorescent unit (Scheme 1). First, silica spheres were prepared using TEOS as the silica source, with CTAB and NaSal as the porous templates. After the templates were removed by HCl/CH_3_OH reflux, mesoporous silica spheres were obtained. After hydrothermal treatment at 160 °C for 12 h, UR and CA were carbonized together and N-CDs formed in the mesoporous silica pores.

**Figure 1:**
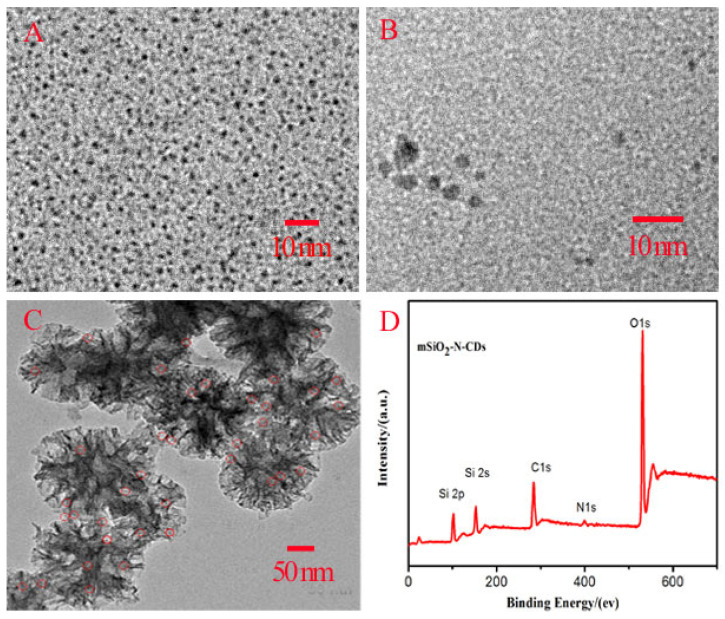
(**A**,**B**) TEM images of NCDs; (**C**) TEM images of mSiO_2_-N-CDs; (**D**) XPS spectrum of mSiO_2_-N-CDs.

Figure 1 presents the TEM view of the N-CDs (Figure 1A,B), mSiO_2_-N-CDs (Figure 1C), and X-ray photoelectron spectroscopy (XPS) of mSiO_2_-N-CDs (Figure 1D). Figure 1A,B indicates that N-CDs are composed of a large number of black spherical dots with a diameter of approximately 2–4 nm. From Figure 1C, it can be seen that the obtained mSiO_2_-N-CDs possess a regular spherical appearance with a diameter of approximately 150 nm and good dispersion. By comparing the light and dark area of the mSiO_2_-N-CDs, it could be determined that the nanospheres are not solid structures and contain many stripe-like channels, which are suitable for embedding N-CDs. As shown in the red circles, some dark spots appeared in the pore channels, with very similar morphology to that of the spherical N-CDs. The successful embedding of N-CDs in mSiO_2_’s pore channels is further confirmed by X-ray photoelectron spectroscopy (XPS), ultraviolet absorption spectroscopy, FTIR spectroscopy, and fluorescence spectroscopy.

The XPS spectrum is presented in Figure 1D. It contains four peaks at 104.3, 154.7, 282.5, 399.8, and 531.7 eV, corresponding to Si 2p, Si 1s, C1s, N1s, and O1s, respectively. This indicates that the probe is composed of C, N, O, and Si, further proving the successful embedding of N-CDs in mSiO_2_. The high-resolution XPS peaks of C1s, N1s and O1s are all presented in Figure S2A–C. The C1s’ spectrum is divided into three peaks at C–C/C=C (281.8 eV), C–N/C–O (283.4 eV), and C=N/C=O (288.3 eV), as shown in Figure S2A. From Figure S2B, it can be seen that the N1s are separated into C-N-C (398.67 eV), O=C-N (399.29 eV), and N-H (401.67 eV). The presence of O=C (530.48 eV) and C-O-C/C-OH (532.62 eV) can be confirmed by Figure S2C.

**Figure 2:**
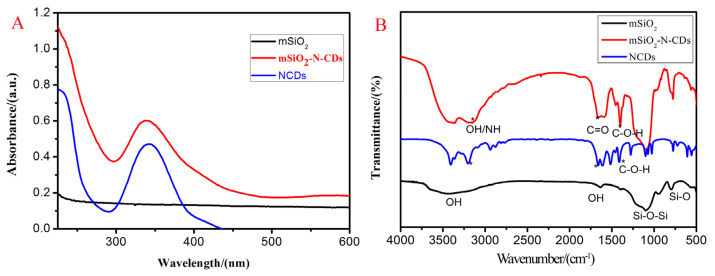
(**A**) UV–VIS spectra of the obtained samples; (**B**) FTIR spectra of the obtained samples.

Figure 2A shows the UV–VIS spectra of the obtained samples, including mSiO_2_ (black curve), NCDs (blue curve), and mSiO_2_-N-CDs (red curve). There is no obvious absorption peak in the black curve, while the absorption peak at 350 nm that appear in the blue curve can be attributed to the π→π* transition of the N-CDs [48]. In the red curve, the absorption peaks are consistent with pure N-CDs, thus further confirming that N-CDs were embedded successfully in the channels of the mesoporous silica. Figure 2B shows the FTIR spectra of the prepared samples. In the black curve (mSiO_2_), the absorption band near 3420 cm^−1^ is determined as the tensile vibration of -OH, the peak at 1625 cm^−1^ is caused by the bending vibration of H-O-H, the two strong peaks at 1080 and 796 cm^−1^ are due to the asymmetric and symmetric stretching of Si-O-Si, respectively, and the peak at 965 cm^−1^ corresponds to the symmetric stretch of Si-OH. Thus, the black curve is consistent with pure mesoporous silica [41]. The blue curve represents N-CDs, and in the red curve (mSiO_2_-N-CDs), as well as the characteristic peaks of mesoporous silica, an absorption band corresponding to -NH_2_ appears near 3210 cm^−1^, a strong characteristic peak of C=O shows around 1679 cm^−1^, and the deformation vibration peak of -OH in -COOH shows at 1386 cm^−1^. These results indicate that N-CDs with abundant amino and carboxyl groups were successfully embedded in the mesoporous silica.

### 3.2. Photoluminescent Properties of mSiO_2_-N-CDs

**Figure 3:**
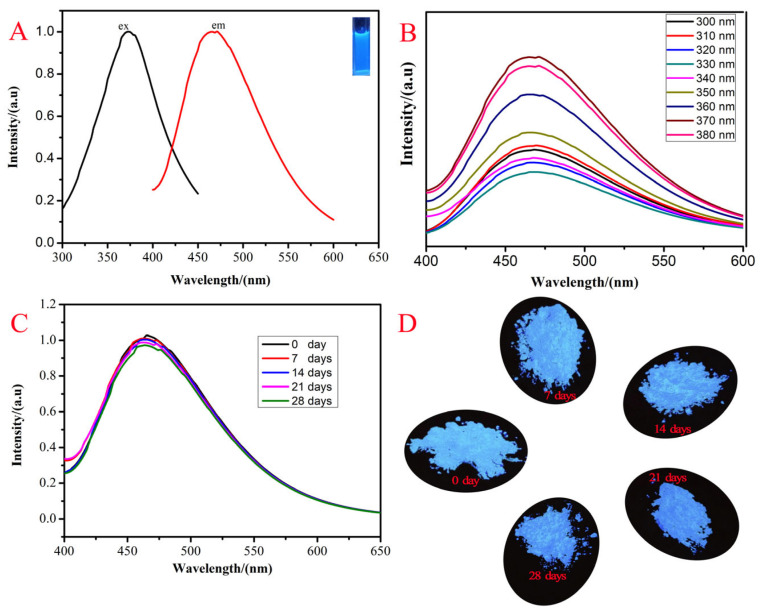
(**A**) The PL spectra of mSiO_2_-N-CDs (inset: photographs of mSiO_2_-N-CDs dispersed in water); (**B**) the emission spectra of mSiO_2_-N-CDs under different excitation; (**C**) the emission spectra of the solid mSiO_2_-N-CDs over time, up to 28 days; (**D**) photographs of the corresponding mSiO_2_-N-CDs powder over time, up to 28 days under 365 nm UV irradiation.

The photoluminescent (PL) performance of the obtained mSiO_2_-N-CDs dispersed in water (0.25 mg·mL^−1^) were characterized in detail, as shown in Figure 3A,B. The PL spectra show the typical excitation and emission peaks of N-CDs, consisting of a wide excitation peak and a broad emission peak. The best excitation peak is near 370 nm and the highest emission peak is around 465 nm. As can be seen from Figure 3B, the emission peak did not move with the change of the excitation wavelength (300–380 nm); however, the emission intensity varied with the excitation wavelength and the optimal excitation wavelength is 370 nm. Unlike many previous reports on CDs [48,49], mSiO_2_-N-CDs exhibited a stable emission peak showing no shift with the adjustment of excitation wavelength, indicating that the size and surface of the prepared mSiO_2_ particles are uniform.

As shown in Figure 3C,D, the solid mSiO_2_-N-CDs produce bright blue fluorescence with a quantum yield (QY) of 32.5% under 365 nm UV irradiation. For further investigation of the fluorescence stability of mSiO_2_-N-CDs, the effect of storage time on the emission intensity was studied. From Figure 3C, it can be seen that the emission intensity of solid mSiO_2_-N-CDs remained almost constant for 28 days (the relative standard deviation was 1.07%). The comparative photos and emission spectra of solid N-CDs and mSiO_2_-N-CDs are presented in Figure S1: mSiO_2_-N-CDs powder emitted strong blue fluorescence under a 365 nm ultraviolet lamp (inset photograph) and solid N-CDs showed much lower fluorescence than that of mSiO_2_-N-CDs due to the ACQ. The stability emission may have been due to the interaction of the amino (-NH_2_) and carboxyl (-COOH) groups on the surface of the N-CDs with the hydroxyl (-OH) groups of the silica pore channels. This interaction effectively avoided ACQ of the N-CDs. Thus, the prepared mSiO_2_-N-CDs possessed stable emission intensity and would be suitable for practical applications.

### 3.3. DA Detection Performance

**Figure 4:**
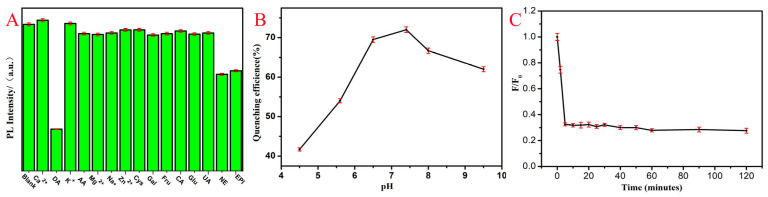
(**A**) Selectivity of mSiO_2_-N-CDs towards various interfering analytes (50 μM); (**B**) the fluorescence quenching efficiency of DA on mSiO_2_-N-CDs at different pH values; (**C**) the effect of culture time on the fluorescence quenching.

The high selectivity of mSiO_2_-N-CDs for the detection of DA was demonstrated by fluorescence intensity tests. Various potentially interfering molecules (including CA, UA, cysteine, glucose, galactose, fructose, epinephrine, and norepinephrine) and ions (Ca^2+^, Zn^2+^, Na^+^, K^+^, and Mg^2+^) were introduced into the mSiO_2_-N-CDs solution (0.25 mg·mL^−1^). The fluorescence intensity of these solutions was then compared with the fluorescence intensity of equivalent solutions with DA added. Here, the concentrations of DA and the interfering analytes were both 50 μM. The results are shown in Figure 4A: Except for epinephrine, norepinephrine, and DA, the fluorescence intensity of mSiO_2_-N-CDs solution did not change significantly. The structures of epinephrine and norepinephrine are similar to that of DA, which resulted in fluorescence quenching. Nevertheless, the fluorescence quenching effect was weaker than that of DA. Additionally, the concentration of epinephrine (<300 pg·mL^−1^) and norepinephrine (<400 pg·mL^−1^) in blood serum is much lower than that of DA [49,50]. Therefore, these data indicate the specificity and selectivity of the mSiO_2_-N-CDs nanoprobe for DA detection.

After verifying the high selectivity and specificity of the probe for DA detection, the experimental conditions, including environmental pH value and reaction time, were optimized. Figure 4B shows the change in the emission intensity of mSiO_2_-N-CDs with DA (DA concentration 50 μM) at different pH values (pH 4–9). The results showed that the fluorescence quenching rate was optimal at pH 7.4.

Consequently, pH 7.4 and room temperature were chosen as the conditions for the detection of DA and the effect of reaction duration on the detection performance was explored. Figure 4C shows that the emission intensity of the original mSiO_2_-N-CDs solution was relatively high, then decreased significantly within 5 min after the addition of DA. This meant that the interaction between mSiO_2_-N-CDs and DA was relatively fast. Furthermore, the emission intensity remained stable when the incubation time was extended to 120 min. In subsequent experiments, a reaction time of 5 min was selected. Therefore, a rapid and simple method for detecting DA was established.

**Figure 5:**
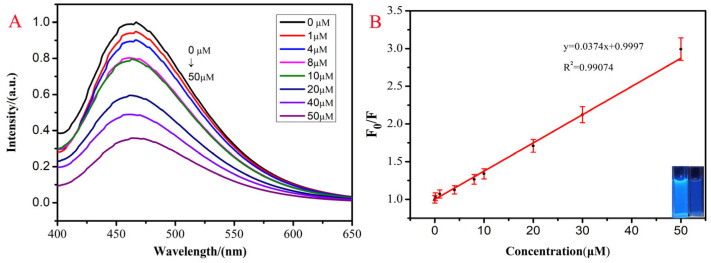
(**A**) Fluorescence emission spectra of mSiO_2_-N-CDs probe in PBS buffer solution (pH = 7.4) with different amounts of DA under 370 nm excitation; (**B**) linear relationship between fluorescence intensity ratio (F_0_/F) and concentration of DA under 370 nm excitation (Inset: the photographs of mSiO_2_-N-CDs solution with DA (50 μM) and without DA under 365 nm ultraviolet lamp).

Under the optimal conditions, the performance of the mSiO_2_-N-CDs probe for DA analysis was determined by adding DA of different concentrations. The corresponding fluorescence spectrum is shown in Figure 5A. As the DA concentration increased, the blue fluorescence of the solution gradually weakened. From Figure 5B, a good linear relationship between F/F_0_ and the concentration of DA was obtained: F/F_0_ = 0.03743c + 0.99974 (R^2^ = 0.99074, with detection limit of 107 nM and linear range of 0–50 µM). The detection limit was calculated based on the equation of D = 3σ/K, in which σ is the standard deviation and K is the slope of the calibration line. The inset in Figure 5B shows photographs of the mSiO_2_-N-CDs solution with (50 μM) and without DA under a 365 nm ultraviolet lamp. Moreover, the repeatability of DA detection by this probe was studied. The mSiO_2_-N-CDs solutions (0.25 mg/mL) were prepared three times to measure a DA solution of 10 µM, and the relative standard deviation (RSD) was 1.55%, indicating that the probe had good repeatability. Table 1 lists the detection range and detection limit of several DA detection sensors, and it can be seen that the obtained mSiO_2_-N-CDs had considerable sensitivity compared with the previously reported sensors.

### 3.4. Real Sample Detection

The urine DA content of healthy volunteers was detected by adding standard DA, as shown in Table 2. The experimental detection results indicate that the recovery rates of different samples range from 98.0% to 100.8%. The relative standard deviation (RSD) was between 0.71% and 2.0%. These satisfactory results show that this method could be feasible for the analysis of actual samples.

### 3.5. Possible Mechanism

**Figure 6:**
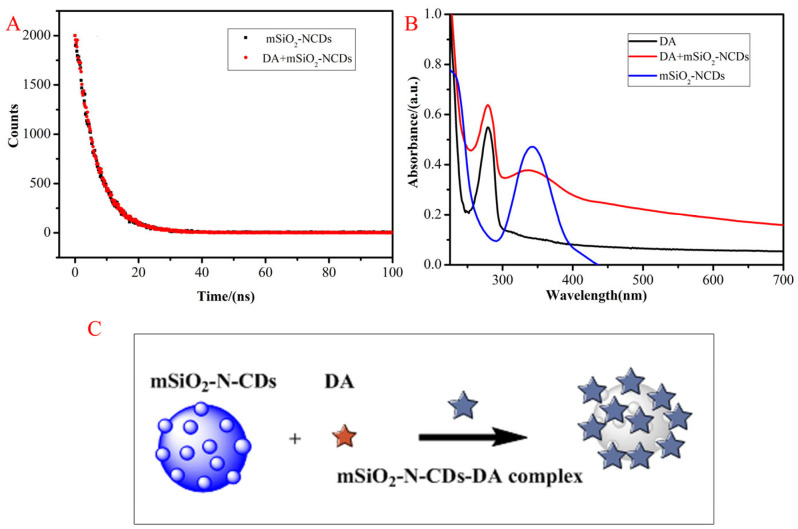
(**A**) Representative lifetime decay profiles of mSiO_2_-N-CDs in the absence (black line) and presence (blue line) of DA; (**B**) UV-visible absorption spectra of mSiO_2_-N-CDs, DA, and mSiO_2_-N-CDs with DA; (**C**) the possible quenching mechanism diagram.

Usually, the fluorescence quenching process includes two types: dynamic quenching and static quenching [53]. The possible quenching mechanism of mSiO_2_-N-CDs on DA was analyzed using fluorescence lifetime decay analysis and UV–VIS spectra. Fluorescence lifetime decay analysis was performed on mSiO_2_-N-CDs with and without DA. As shown in Figure 6A, the average lifetime of the mSiO_2_-N-CDs sensor (τ_0_) was 6.43 ns. After the addition of DA (10 μM), the lifetime (τ_1_) was 6.45 ns; that is, the ratio value of τ_0_/τ_1_ was about equal to 1. According to previous reports [54,55], if τ_0_/τ_1_ = 1, static quenching occurs. This indicates that the fluorescence quenching mechanism may be static quenching.

The quenching rate constant K_q_ can be calculated through the Stern–Volmer equation (F_0_/F = 1 + K_SV_[Q] = 1 + K_q_τ_0_[Q]). Here, F_0_ represents the emission intensity at 465 nm without DA, F represents the emission intensity with the addition of DA, K_SV_ is the Stern–Volmer quenching constant, τ_0_ is the lifespan without DA, and [Q] is the concentration of DA. From the calculation, it was concluded that the value of k_q_ was 3.8 × 10^12^ M^−1^·s^−1^, much greater than 2.0 × 10^10^ M^−1^·s^−1^. This result further implied the existence of static quenching [55].

Figure 6B presents the UV–VIS spectra of mSiO_2_-N-CDs, DA, and mSiO_2_-N-CDs with DA. The absorption peak of mSiO_2_-N-CDs, appearing around 350 nm, was assigned to the n-π* electron transition of the C=O and C-N groups of the CDs [48]. The typical absorption of π-orbitals in aromatic rings resulted in a sharp peak at 280 nm for DA [48]. When DA was added into the mSiO_2_-N-CDs solution, the absorption band around 350 nm was significantly weaker and a new wide absorption band (450–600 nm) was generated, implying the formation of a new complex (mSiO_2_-N-CDs-DA) between mSiO_2_-N-CDs and DA [56,57]. This might be attributed to the forces between mSiO_2_-N-CDs and DA, such as the hydrogen bonding and the π-π conjugation between them, due to the abundant amino (-NH_2_) and carboxyl (-COOH) groups on the surface of mSiO_2_-N-CDs, and the amino (-NH_2_) and hydroxyl (-OH) groups on the DA molecule. This result supports the static quenching mechanism (as depicted in Figure 6C) we proposed.

## 4. Conclusions

An mSiO_2_-N-CDs fluorescence probe was successfully prepared for DA detection via a one-pot hydrothermal reaction using CA as the carbon source, UR as the nitrogen source and mSiO_2_ as the skeleton. N-CDs were generated in situ in the pore channels of mesoporous silica. Due to the immobilization of the N-CDs by the mesoporous silica pores, the self-quenching caused by the aggregation of N-CDs was effectively avoided. Furthermore, solid-state mSiO_2_-N-CDs exhibited stable PL with a high quantum yield (32.5%).

Based on the high selectivity and sensitivity of mSiO_2_-N-CDs for DA, a rapid, simple, and sensitive DA detection method was established, with a detection limit of 107 nM and a linear range of 0–50 µM. This probe was applied in the determination of the DA content in actual urine samples, with the spiked recovery ranging from 98.0% to 100.8% and the RSD from 0.71% to 2.0%. Due to the simple and convenient preparation process of the mSiO_2_-N-CDs, their good fluorescence stability, and their high sensitivity and selectivity for DA, the developed fluorescent probe has potential applications in determining the DA content in real biological samples.

## Figures and Tables

**Table 1:** Comparison of fluorescence sensors for detection of DA.

Method	Probe	Liner Range (μM)	LOD (μM)	Reference
FL/quenching	Au@N-GQD	0.0–80.0	0.52	[25]
FL/quenching	F-CuInS_2_	0.5–40.0	0.2	[26]
FL/quenching	S,N-CQD	0–50	0.082	[27]
FL/quenching	Fe-CQDs	0.01–50	0.005	[28]
FL/quenching	N-CQDs	0.01–200	0.05	[29]
FL/quenching	r-QDs@SiO_2_	10.0–80	0.12	[51]
FL/quenching	GQDs	0.25–50	0.09	[52]
FL/quenching	mSiO_2_-N-CDs	0–50	0.107	This work

**Table 2:** Detection of DA in human urine sample.

Sample	Added (µM)	Found (µM)	Recovery/%	RSD%, *n* = 3
1	0.5	0.49	98.0	0.71
2	1	0.99	99.0	1.80
3	5	5.04	100.8	2.0
4	40	40.30	100.8	2.0

## Data Availability

The data presented in this study are available on request from the corresponding author.

## Supplementary Materials

The following supporting information can be downloaded at: https://www.mdpi.com/article/10.3390/nano13232989/s1, Figure S1: comparative PL of solid N-CDs and solid mSiO_2_-N-CDs, Figure S2: (A) C1s spectrum of mSiO_2_-N-CDs (B) N1s spectrum of mSiO_2_-N-CDs (C) O1s spectrum of mSiO_2_-N-CDs.
