# Supplementary material for: One-Pot Hydrothermal Synthesis of mSiO2-N-CDs with High Solid-State Photoluminescence as a Fluorescent Probe for Detecting Dopamine
Source: Nanomaterials (Basel). 2023 Nov 21;13(23):2989. doi: 10.3390/nano13232989 (PMC10708435; doi:10.3390/nano13232989)
Supplement: Supplementary file 1 [file nanomaterials-13-02989-s001.zip › nanomaterials-2708048-supplementary.pdf]

Supporting Material

One-pot hydrothermal synthesis of $mSiO_2$-N-CDs with high solid-state photo -luminescence as a fluorescent probe for detecting dopamine

Xiaogao Guan[1] , Xianzhu Xu[2] , Yanli Wu[1*], Ruchun Yang[1] , Xi Chen[1], Fangfang Kong[1*] , Qiang Xiao

1 Jiangxi Key Laboratory of Organic Chemistry, Jiangxi Science and Technology Normal University, Nanchang, 330013, China; 18270143800@139.com (X.G.);wanny118@126.com (Y.W.); ouyangruchun@163.com (R.Y.); chenxi2016@email.ncu.edu.cn (X.C.); xiaoqiang@tsinghua.org.cn(Q.X.)

2 College of Life Science, Jiangxi Normal University, Nanchang 330031, China; 9901189@163.com (X.X.);

* Correspondence:wanny118@126.com(Y.W.); fkingcn@163.com (F.K.);Tel: (+86-0791-83805183)

Chemicals and Instruments

Tetraethyl orthosilicate (TEOS, 99.0 %) and cetyltrimethylammonium bromide (CTAB, 99%) were purchased from Sigma-Aldrich. Sodium salicylate (NaSal, 99.8%) was purchased from Shanghai Haohong Scientific Co., Ltd. Citric acid (CA, 99.5%) was purchased from Hefei Qiansheng Biotechnology Co., Ltd. All other reagents were of analytical grade and used as received without further purification.

The morphology and particle size of the samples were observed by field emission high-resolution transmission electron microscopy (TEM; Tecnai G20, 200 kV). X-ray photoelectron spectroscopy (XPS) was performed with AXIS SUPRA spectrometer. The composition of the samples was determined by infrared spectrometry (Perkin- Elmer C99957) in a test range of 500 to 4000 cm^{-1}. Excitation and emission spectra were obtained using a Hitachi F7000 fluorescence spectrometer. UV-VIS spectra were measured using an Agilent 8453 UV-VIS spectrometer. Fluorescence decay curves were determined using a Hamamatsu

C11367 fluorescence lifetime spectrometer. Absolute photoluminescence (PL) quantum yields were determined using a Hamamatsu system (type C11347).

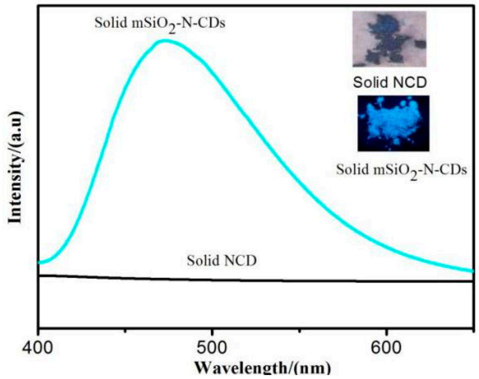

Figure S1. comparative PL of solid NCD (black line) and solid $mSiO_2$-N-CDs (blue line) ; inset: photos of solid NCD and solid $mSiO_2$-N-CDs under 365 nm UV lamp

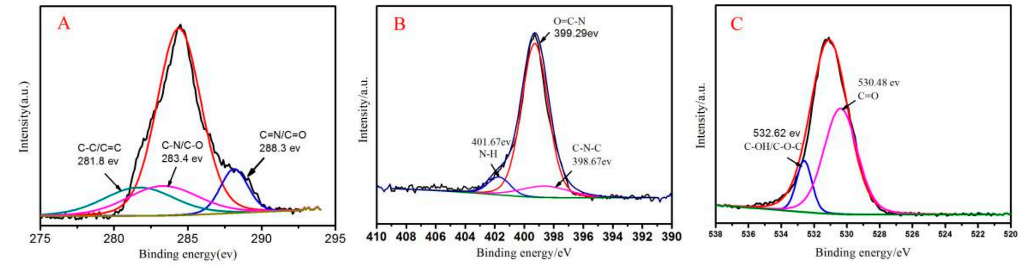

Figure S2. (**A**) C1s spectrum of $mSiO_2$-N-CDs (**B**) N1s spectrum of $mSiO_2$-N-CDs (**C**) O1s spectrum of $mSiO_2$-N-CDs
